# Muscle Mass Index Decline as a Predictor of Lung Function Reduction in the General Population

**DOI:** 10.1002/jcsm.13663

**Published:** 2024-12-17

**Authors:** Joon Young Choi, Chin Kook Rhee, Sang Hyuk Kim, Yong Suk Jo

**Affiliations:** ^1^ Department of Internal Medicine, Division of Pulmonary and Critical Care Medicine, College of Medicine, Incheon St. Mary's Hospital The Catholic University of Korea Seoul Republic of Korea; ^2^ Department of Internal Medicine, Division of Pulmonary and Critical Care Medicine, College of Medicine, Seoul St. Mary's Hospital The Catholic University of Korea Seoul Republic of Korea; ^3^ Department of Internal Medicine, Division of Pulmonary, Allergy, and Critical Care Medicine, Dongguk University Gyeongju Hospital Dongguk University College of Medicine Gyeongju Republic of Korea

**Keywords:** exacerbation, general population, lung function, muscle mass index

## Abstract

**Background:**

This study explores the link between muscle mass decline and lung function deterioration, which can worsen respiratory health by reducing exercise capacity and quality of life. The relationship between muscle mass index (MMI) changes and lung function in the general population remains unclear, especially as muscle mass fluctuates with aging. We aimed to clarify this dynamic relationship by examining how changes in muscle mass impact pulmonary function and the development of respiratory symptoms.

**Methods:**

We utilized the Ansan and Ansung Cohort Study of the Korean Genome and Epidemiology Study (KoGES) database, a large‐scale prospective cohort, enrolling participants aged 40 to 69 years with lung function and body composition measurements. Over 12 years, data were collected biannually. The study assessed associations between changes in MMI and lung function trends, with cT1‐T3 calculated using the linear regression coefficient and stratified by tertile. Survival analysis was then performed to examine differences in time to first airflow obstruction (AFO) and exacerbation among the tertiles.

**Results:**

A total of 2956 participants were enrolled in this study. At baseline, participants with higher MMI tended to be younger, had fewer co‐morbidities and exhibited better lung function. Those with a steeper MMI decline rate exhibited a more rapid forced expiratory volume in 1 s (FEV_1_) decline over a 12‐year follow‐up (cT1: 43.3 mL/year, cT2: 38.4 mL/year, cT3: 33.2 mL/year, *p* < 0.001). Forced vital capacity (FVC) decline were more pronounced in groups with greater MMI decline rates (cT1: 38.5 mL/year, cT2: 32.8 mL/year, cT3: 26.0 mL/year, *p* < 0.001). Although, the time to first AFO did not differ significantly among T1‐T3 groups, the time to first exacerbation related to wheezing event was significantly lower in cT3 group than in cT1 group (HR: 0.786, 95% CI: 0.629, 0.982).

**Conclusions:**

A faster decline in MMI was associated with more rapid decline of both FEV_1_ and FVC and a higher risk of developing exacerbations of respiratory symptom. Although AFO was not associated with changes in MMI, further research is needed to explore the long‐term relationships between muscle mass and the effects of preventive interventions aimed at maintaining muscle mass and respiratory health.

**Abbreviations**
AFO: airflow obstruction
ANOVA: one‐way analysis of variance
ASMI: appendicular skeletal mass index
BMI: body mass index
COPD: chronic obstructive pulmonary disease
CT: computed tomography
FEF25–75: 25% and 75% of vital capacity
FEV1: forced expiratory volume in 1 s
FFMI: fat free mass index
FVC: forced vital capacity
KoGES: Korean Genome and Epidemiology Study
MMI: muscle mass index

## 1 Introduction

Decline in muscle mass and lung function are crucial indicators of deteriorating health, particularly as individuals age. Sarcopenia, the loss of muscle mass and strength, is a well‐recognized consequence of aging, but its implications extend beyond the elderly. The European Working Group of Sarcopenia in Older people (2019) has highlighted the clinical significance of ‘secondary sarcopenia’, which arises from underlying conditions other than aging [1]. The interplay between sarcopenia and lung function decline is multifactorial, involving exposure to toxic agents (e.g., tobacco smoking), reduced anabolic hormone activity, immune system weakening and oxidative stress. These factors contribute to both sarcopenia and the deterioration of lung function [2, 3, 4, 5]. Moreover, the progressive loss of muscle mass impairs exercise tolerance, exacerbates physical disability and diminishes quality of life, thereby accelerating lung function decline [6, 7, 8, 9]. While sarcopenia often associated with aging, it also affects younger individuals experiencing prolonged physical inactivity, chronic illness, or specific medical conditions [1, 7].

Traditional methods like Body mass index (BMI) fail to accurately distinguish between muscle and fat mass, necessitating more precise tools [10]. Bioelectrical impedance analysis (BIA) a is simple and easy tool that enables us to better characterize an individual's nutritional status by measuring body compositions [11]. Several studies have reported that reduced skeletal muscle mass is associated with lower lung function [12, 13]. Fat mass can frequently mask muscle mass reduction. Thus, its effects should be considered together with muscle mass [14]. Fat free mass index (FFMI) correlates with airflow limitation and extent of emphysema. Thus, FFMI is regarded as one of the surrogate markers for muscle mass in chronic obstructive pulmonary disease (COPD) [S1−S3]. Similarly, appendicular skeletal mass index (ASMI) and skeletal muscle mass index (MMI) are other measures associated with impaired functional status in COPD [S4, S5].

Despite evidence linking reduced muscle mass to lower lung function, the longitudinal relationship between dynamic changes in muscle mass and lung function over time remains underexplored. Understanding these temporal dynamics is critical for evaluating how fluctuations in muscle mass impact lung function. This study aims to elucidate the dynamic relationship between muscle mass and pulmonary function in a general population.

## 2 Methods

### 2.1 Study Design and Eligibility Criteria

We used data from the longitudinal, population‐based observational cohort of two population‐based studies (rural Ansung and urban Ansan cohorts) as part of the Korean Genome Epidemiology Study (KoGES). This project recruited the general population aged between 40 and 69 years to assess the incidence and risk factors of various chronic disorders. The initial baseline survey was conducted between 2001 and 2002, and participants were subsequently followed up every 2 years until 2019. During each follow up visit, comprehensive data were collected, including lifestyle characteristics, medical history, subjective symptoms and incidence of diseases, providing a robust dataset for analysing long‐term health trends.

Inclusion criteria were follows: (1) those with spirometric measurement at baseline survey; (2) those with tracking of spirometry over a minimum of three visits; and (3) those with measurements of body compositions. Individuals diagnosed with asthma or COPD and those who were currently under treatment, or cases with unidentifiable diagnostic data were excluded. If they exhibited a forced expiratory volume in 1 s (FEV_1_)/forced vital capacity (FVC) ratio < 0.7 at baseline spirometric measurement, they were also excluded (Figure S1).

### 2.2 Clinical Variables

At baseline assessment, demographic and socio‐economic data including age, sex, smoking history and pack‐years, education level and income were obtained by trained interviewers. Anthropometric parameters such as height and weight, were measured. A protocolized and systematic questionnaire was used to identify medical history at baseline visit and newly developed disease at every visit. The detailed methodological information of the KoGES cohort has been described in a previous cohort profile report [15].

Physical activity was assessed through daily time of moderate or vigorous activities questionnaires. Active physical activity was defined as at least 60 min per day of moderate activity or 30 min per day of vigorous activity [16]. Laboratory test was conducted to investigate general medical conditions.

### 2.3 Body Composition Measurements

Body composition was assessed using a multifrequency BIA (InBody 3.0, Biospace, Seoul, Korea) at baseline visit and biannually during the study period. It assumes the human body as five interconnecting cylinders and performs impedance measurement directly on each compartment at four specific frequencies (5, 50, 250 and 500 kHz) in five segments (both arms and legs, and trunk) using a tetrapolar 8‐point tactile electrode system. Based on the electrical impedance of tissue water, it estimates the amount of body fat, bone and water. Data on muscle mass, body water, fat mass, fat free mass and body protein were obtained from the device.

In this study, total lean body mass was estimated by dividing the body's total water content by 0.73, a method validated by earlier research [17, 18]. For limbs, lean body mass equates to skeletal muscle mass. Appendicular skeletal mass (ASM) was calculated by totalling lean mass in all four limbs [19]. MMI was then derived by dividing ASM by body weight in kilograms and multiplying by 100 as shown in a previous study [20]. BMI was calculated as body weight (kg) divided by the square of the height (m). Waist circumference was manually measured in the standing position at the level of umbilicus.

### 2.4 Lung Function Measurement

Pulmonary function test was performed by a skilled technician using a standard spirometer (Vmax‐2130, Sensor Medics, Yorba Linda, CA, USA). Calibration and quality control of the test were performed regularly based on guidelines [21]. Pre‐bronchodilator values of FEV_1_ and FVC (in litres and percentage of the predicted value), FEV_1_/FVC ratio and forced expiratory flow between 25% and 75% of vital capacity (FEV25–75) were obtained.

### 2.5 Statistical Analysis

All statistical analyses were performed using R software (ver. 4.3.1; R Development Core Team, Vienna, Austria). Continuous variables are expressed as mean ± standard deviation. Categorical variables are presented with numbers and percentages. Clinical, biochemical and body composition measurements were compared between three tertile groups of MMI using One‐way analysis of variance (ANOVA) for continuous variables that followed a normal distribution and the Kruskal‐Wallis H test for variables that did not follow a normal distribution. Categorical variables were assessed using the χ2 test. Tertile groups of MMI were calculated with different cut‐off values for each sex.

The primary outcome of this study was the longitudinal change in lung function, with the secondary outcome being the risk of exacerbation. Longitudinal changes of MMI gradients for 12 years were calculated with linear regression coefficient and stratified with tertile (cT1‐T3) (Figure S2). Linear mixed models were used to evaluate differences of longitudinal lung function decline rates including FEV_1_, FVC, FEV_1_/FVC and FEF25–75 between cT1‐T3 groups for 12 years. Covariates including age, sex, BMI, smoking history, socio‐economic status (including education and income level), respiratory symptoms (dyspnoea, wheeze, cough and sputum) and radiologic abnormalities (emphysema, interstitial lung abnormalities and bronchiectasis) were adjusted.

Survival analysis was conducted to evaluate time to airflow obstruction (AFO) defined by FEV1/FVC < 0.7 among tertiles of MMI decline rates (cT1‐cT3) and time to first exacerbation. Exacerbation was defined by development of symptoms such as wheezing or dyspnoea within 12 months, not necessarily requiring hospitalization. The effects of changes in MMI on outcomes were assessed using Cox proportional hazards analysis. Model 1 adjusted for age, sex, BMI and smoking status. Model 2 added physical activity and FEV_1_. Model 3 included metabolic syndrome, and model 4 further adjusted for radiologic abnormalities (emphysema, interstitial lung abnormalities and bronchiectasis) for the exacerbation analysis, and respiratory symptoms were additionally adjusted for the AFO analysis.

## 3 Results

### 3.1 Baseline Characteristics

Table 1 presents baseline characteristics across distinct MMI categories denoted as T1, T2 and T3. A total of 2956 participants were included in the analysis. There was a significant age difference across MMI categories (T1: 53.3 ± 8.5 years, T2: 51.0 ± 8.2 years, T3: 49.5 ± 8.0 years, *p* < 0.001). There was no difference in gender distribution. Educational attainment also showed a gradient, with lower levels prevalent in T1 (elementary school or lower: 32.0%) than in T3 (university or above: 16.4%, *p* < 0.001). Daily active physical activity showed significant differences among groups (36.7% in T1, 42.4% in T2, and 46.7% in T3, *p* < 0.001). Smoking status revealed a decreasing trend in non‐smokers (T1: 67.0%, T2: 66.8%, T3: 65.6%, *p* = 0.026). There was a corresponding reduction in pack‐years across T1 to T3 (T1: 22.6 ± 17.7, T2: 20.8 ± 14.9, T3: 18.9 ± 13.6, *p* = 0.014). Several co‐morbidities, including hypertension, diabetes mellitus and coronary artery disease, displayed a significant decreasing trend across categories. Lung function measures including FVC, FEV_1_ and FEF25–75 exhibited significant increasing trends across categories (*p* < 0.001).

**TABLE 1:** Baseline characteristics by muscle mass index (MMI) categories.

	Overall (*N* = 2956)			
	T1 (*N* = 941)	T2 (*N* = 1009)	T3 (*N* = 1006)	*p* for trend
Age, years	53.3 ± 8.5	51.0 ± 8.2	49.5 ± 8.0	< 0.001
Sex, male	395 (42%)	406 (40.2%)	399 (39.7%)	0.560
Area, urban (vs. rural)	684 (72.7%)	745 (73.8%)	691 (68.7%)	0.027
Marriage state, unmarried	92 (9.8%)	79 (7.8%)	60 (6%)	0.007
Educational level				< 0.001
Elementary school or lower	301 (32.0%)	257 (25.6%)	237 (23.6%)	
Middle or high school	495 (52.7%)	579 (57.7%)	602 (60%)	
University or above	144 (15.3%)	168 (16.7%)	164 (16.4%)	
Income (KRW), < 2 000 000	568 (61.4%)	577 (57.9%)	585 (58.8%)	0.548
Nutrition				
Total energy, kcal	1929.8 ± 669.4	1925.4 ± 691.5	1938.4 ± 658.3	0.909
Protein, g	66.3 ± 28.1	67.1 ± 33.7	66.1 ± 26.4	0.749
Physical activity (active)	340 (36.7%)	423 (42.4%)	461 (46.7%)	< 0.001
Smoking status				
Non‐smoker	625 (67.0%)	671 (66.8%)	656 (65.6%)	0.026
Current or former smoker	308 (33.0%)	333 (33.1%)	344 (34.4%)	
Current	153 (16.4%)	183 (18.2%)	214 (21.4%)	
Former	155 (16.6%)	150 (14.9%)	130 (13.0%)	
Smoking, pack‐years	22.6 ± 17.7	20.8 ± 14.9	18.9 ± 13.6	0.014
Co‐morbidity				
Hypertension	306 (32.5%)	229 (22.7%)	148 (14.7%)	< 0.001
Diabetes mellitus	111 (11.8%)	112 (11.1%)	73 (7.3%)	0.001
Coronary artery disease	16 (1.7%)	13 (1.3%)	4 (0.4%)	0.019
Congestive heart failure	4 (0.4%)	2 (0.2%)	1 (0.1%)	0.320
Dyslipidaemia	45 (4.8%)	35 (3.5%)	28 (2.8%)	0.059
Chronic Kidney disease	45 (4.8%)	31 (3.1%)	35 (3.5%)	0.119
Cerebrovascular disease	18 (1.9%)	10 (1%)	12 (1.2%)	0.183
Lung function				
FVC, L	3.4 ± 0.8	3.6 ± 0.8	3.7 ± 0.8	< 0.001
FVC, %predicted	102.9 ± 13.1	104.9 ± 12.9	106.4 ± 13.6	< 0.001
FEV_1_, L	2.8 ± 0.6	2.9 ± 0.6	3.1 ± 0.7	< 0.001
FEV_1_, % predicted	112.9 ± 16.1	114.3 ± 15.2	115.1 ± 15.3	0.007
FEV_1_/FVC	81.7 ± 5.1	81.8 ± 5.1	81.8 ± 5.5	0.901
FEF25–75	3.1 ± 1.0	3.2 ± 1.0	3.3 ± 1.0	< 0.001
FEF25–75, % predicted	109.1 ± 31.0	110.2 ± 29.7	109.5 ± 28.8	0.702
Radiologic findings				
Emphysema	12 (3%)	12 (2.5%)	18 (4%)	0.434
Interstitial lung abnormality	14 (3.5%)	23 (4.8%)	12 (2.7%)	0.236
Bronchiectasis	15 (3.8%)	29 (5.9%)	18 (4%)	0.223

*Note:* Data are presented as number (%) or mean ± standard deviation (SD).

Abbreviations: FEF, forced expiratory flow; FEV_1_, forced expiratory volume in 1 s; FVC, forced vital capacity; KRW, Korean won.

### 3.2 Body Compositions and Metabolic Parameters

Table S1 delineates biochemical and body composition measurements stratified by MMI tertiles. The analysis of muscle mass revealed no significant differences across tertiles. Conversely, significant decreasing trends were evident for fat mass, percentage body fat, BMI and waist circumference across ascending MMI tertiles (all *p* < 0.001). Furthermore, several metabolic parameters, including fasting glucose, blood pressure and triglycerides, displayed noteworthy decreasing trends across MMI tertiles (all *p* < 0.001). The prevalence of metabolic syndrome also exhibited a consistent decline, decreasing from 51.9% in T1 to 16.8% in T3 (*p* < 0.001).

### 3.3 Longitudinal Changes of Lung Function Parameters by MMI Categories

**FIGURE 1:**
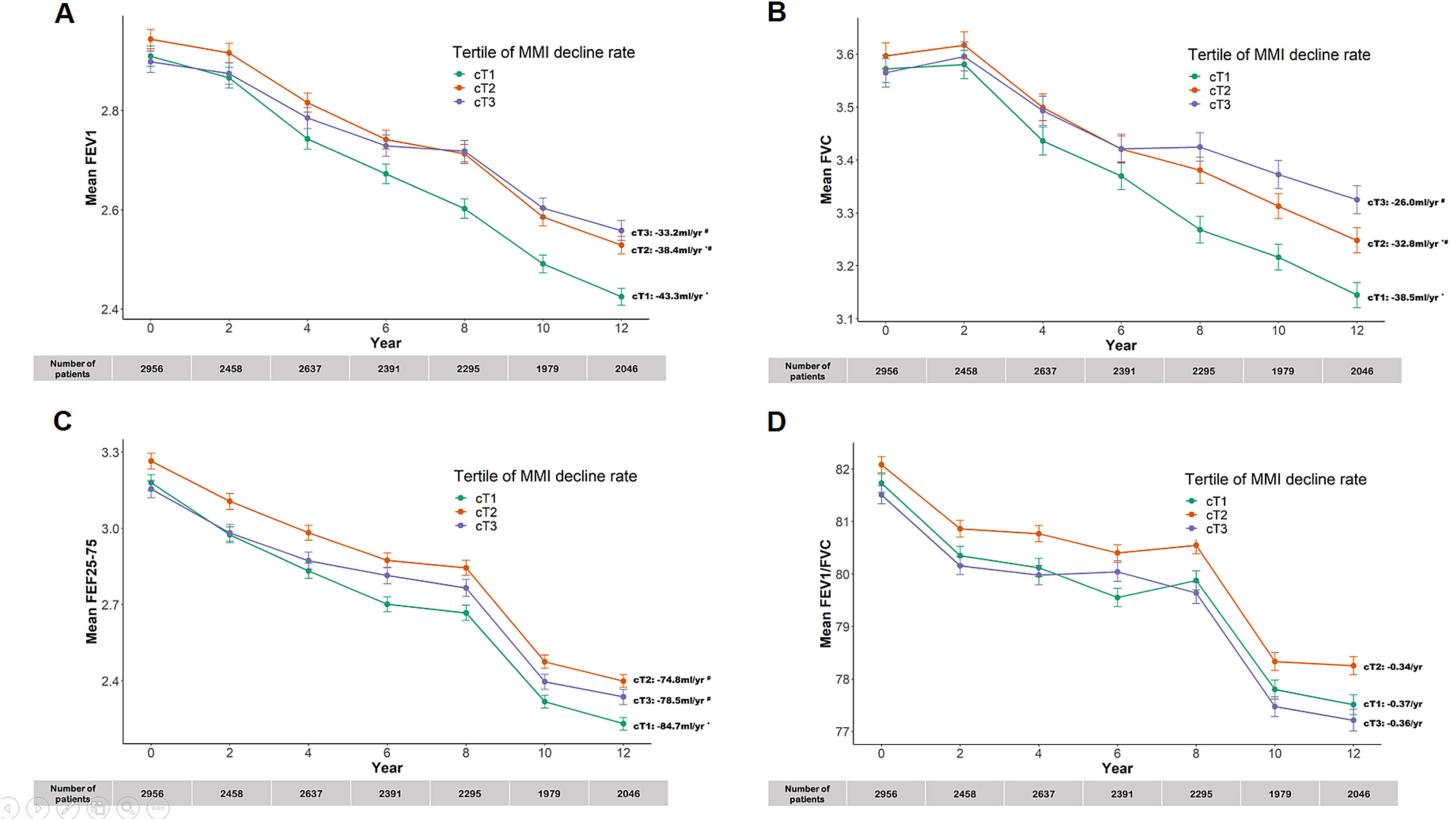
Longitudinal trend of lung function by MMI decline rate. (A) FEV_1_ showed a decline of −43.3 mL/year (95% CI: [−44.7, −42.0]) for cT1, −38.4 mL/year (95% CI: [−40.6, −36.1]) for cT2 (*p* < 0.05 compared with cT1 and cT3), and −33.2 mL/year (95% CI: [−35.7, −30.8]) for cT3 (*p* < 0.05 compared with cT1). (B) FVC decreased by −38.5 mL/year (95% CI: [−40.2, −36.8]) in cT1, −32.8 mL/year (95% CI: [−35.6, −30.0]) in cT2 (*p* < 0.05 compared with cT1 and cT3), and −26.0 mL/year (95% CI: [−29.0, −22.9]) in cT3 (*p* < 0.05 compared with cT1). (C) FEF25–75 decreased by −84.7 mL/year (95% CI: [−88.5, −81.0]) in cT1, −74.8 mL/year (95% CI: [−84.5, −72.0]) in cT2 (*p* < 0.05 compared with cT1), and −78.5 mL/year (95% CI: [−81.5, −68.0]) in cT3 (*p* < 0.05 compared with cT1). (D) The FEV_1_/FVC ratio decreased by −0.37/year (95% CI: [−0.39, −0.34]) in cT1 and −0.34/year (95% CI: [−0.38, −0.30]) in cT2. It decreased by −0.36/year (95% CI: [−0.40, 0.31]) in cT3 (all *p* ≥ 0.05). All changes in lung function were analysed with adjustments for age, sex, BMI, education and income level and smoking status. *vs. cT1 *p* < 0.05; ^#^vs. cT3 *p* < 0.05. BMI, body mass index; FEF, forced expiratory flow; FEV_1_, forced expiratory volume in 1 s; FVC, forced vital capacity; MMI, muscle mass index.

Figure 1 illustrates longitudinal trends of lung functions across different MMI decline rate categories. FEV_1_ showed a decline of −43.3 mL/year for cT1, −38.4 mL/year for cT2 (*p* < 0.05 compared with cT1 and cT3), and −33.2 mL/year for cT3 (*p* < 0.05 compared with cT1). FVC decreased by −38.5 mL/year in cT1, −32.8 mL/year in cT2 (*p* < 0.05 compared with cT1 and cT3), and −26.0 mL/year in cT3 (*p* < 0.05 compared with cT1). FEF25–75 decreased by −84.7 mL/year in cT1, −74.8 mL/year in cT2 (*p* < 0.05 compared with cT1), and −78.5 mL/year in cT3 (*p* < 0.05 compared with cT1). The FEV_1_/FVC ratio decreased by −0.37/year in cT1 and −0.34/year in cT2. It decreased by −0.36/year in cT3 (all *p* ≥ 0.05).

An additional analysis of lung function changes was conducted by categorizing participants into two groups based on whether they showed an increase or decrease in MMI over the study period (slope of MMI trajectory ≥ 0 vs. < 0, as shown in Figure S3). Individuals with an increase in MMI experienced a slower decline in FEV_1_, FVC and FEF25–75.

### 3.4 Risk of AFO According to the Decline Rate of MMI Categories

To figure out the association between the decline rate of MMI and AFO, four models were employed, each adjusted for varying sets of covariates. Compared with the cT1 group, there was no significant difference in the risk of time to AFO cT2 and cT3. (hazard ratio (HR) 0.809, 95% confidence interval (CI): 0.615, 1.064 for cT2 and HR 1.124, 95% CI: 0.863, 1.466 for cT3 group). The analysis of the decline rate of MMI categories by tertile did not reveal any significant correlation with the time to AFO even after adjusting covariates (Table S2 and Figure S4).

An additional analysis of the risk of AFO based on whether individuals showed an increase or decrease in MMI over the study period indicated that changes in MMI did not significantly affect the time to AFO (Table S3).

### 3.5 Risk of Exacerbation According to Decline Rate of MMI Categories

**FIGURE 2:**
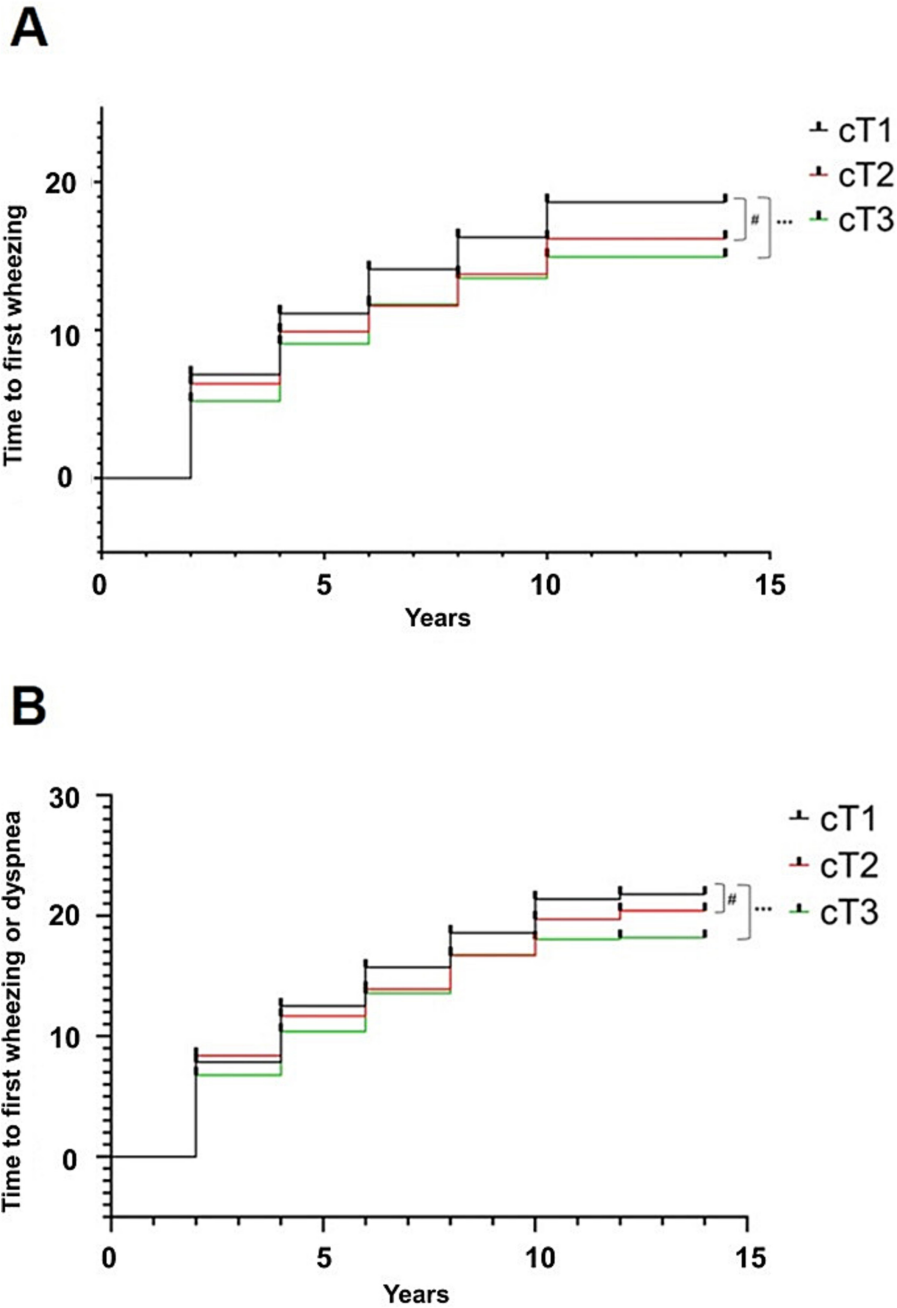
The Kaplan–Meier curves for the onset of (A) wheezing and (B) wheezing or dyspnoea, stratified by MMI decline rate. (a) ^#^ *p* = 0.142; ***p = 0.034 and (b) ^#^ *p* = 0.504; ***p = 0.093.

Investigation into the risk of exacerbation revealed a discernible trend of diminished risk in subsequent comparison of the decline rate of MMI categories, cT2 and cT3, relative to cT1. For wheezing, a significant risk reduction was observed in the cT3 group, demonstrating that more significant MMI decline correlates with lower risk of wheezing development. Notably, the risk of wheezing was reduced approximately 14% in cT2 group and 21% in cT3 group. However, this trend is less pronounced when considering wheezing or dyspnoea together (Figure 2). Adjustments across different models demonstrated the consistency of these results (Table 2), and sensitivity analysis also showed that an increase in MMI was associated with a delayed time to the first exacerbation (Table S4).

**TABLE 2:** Time to first exacerbation.

MMI decline rate	Hazard ratio (95% Confidence interval)				
	Crude HR	Model 1	Model 2	Model 3	Model 4
**Wheezing**					
cT1	1 (reference)	1 (reference)	1 (reference)	1 (reference)	1 (reference)
cT2	0.856 (0.695–1.053)	0.877 (0.711–1.082)	0.876 (0.710–1.082)	0.889 (0.720–1.098)	0.801 (0.589–1.091)
cT3	0.786 (0.629–0.982)	0.793 (0.633–0.993)	0.752 (0.599–0.945)	0.768 (0.611–0.966)	0.662 (0.463–0.947)
**Wheezing or dyspnoea**					
cT1	1 (reference)	1 (reference)	1 (reference)	1 (reference)	1 (reference)
cT2	0.931 (0.753–1.150)	0.959 (0.774–1.186)	0.965 (0.778–1.120)	0.974 (0.785–1.121)	0.830 (0.610–1.129)
cT3	0.821 (0.652–1.034)	0.827 (0.655–1.045)	0.806 (0.637–1.022)	0.811 (0.640–1.029)	0.613 (0.423–0.887)

*Note:* Model 1: adjusted for age, sex, BMI and smoking status. Model 2: adjusted for age, sex, BMI, smoking status, physical activity and FEV_1_. Model 3: adjusted for age, sex, BMI, smoking status, physical activity, FEV_1_ and metabolic syndrome. Model 4: adjusted for age, sex, BMI, smoking status, physical activity, FEV_1_, metabolic syndrome and radiologic abnormalities (emphysema, interstitial lung abnormalities and bronchiectasis).

Abbreviations: BMI, body mass index; FEV_1_, forced expiratory volume in 1 s; MMI, muscle mass index.

## 4 Discussion

This study addresses an important relationship between dynamic changes in muscle mass and lung function decline. As aging becomes a prevailing challenge, decline in muscle mass (termed sarcopenia) and deteriorating lung function are intertwined. Secondary sarcopenia, not solely attributed to aging, but influenced by factors such as tobacco smoking, hormonal changes and oxidative stress, has gained interests as its significant effects on overall human health has gained recognition. Additionally, this study highlights how progressive muscle mass loss can contribute to reduced lung function and development of respiratory symptom. Gradual loss of muscle mass with lower lung function might lead to limited exercise tolerance and lower quality of life, which can impact not only the elderly, but also individuals of all ages facing physical inactivity or chronic illness.

There are few studies on the longitudinal association between sarcopenia and chronic airway diseases, such as asthma and COPD, and this relationship has been documented across all levels of disease severity, and some studies have also linked it to an increased risk of mortality [22, 23, 24, 25, 26, 27]. However, there is a lack of research involving the general population, and the clear mechanism by which sarcopenia influences the development of chronic airway diseases has not yet been clearly elucidated. Low muscle mass is often associated with aging and increased mortality in the general population [28, 29, 30]. A reduction in respiratory muscle mass and strength, including the diaphragm and intercostal muscles, can make it harder for the lungs to fully expand, thereby limiting airflow. Additionally, as sarcopenia progresses, fat infiltration into muscle tissue often increases, leading to a rise in fat mass. This not only further weakens the muscles but also contributes to inflammation, atrophy fibrosis, which can reduce the elasticity of lung tissues and further impair lung function [31, 32, 33, 34]. In an asthma model mice study, miR‐133a, primarily known for its expression in skeletal muscle and cardiac myocytes, has also been observed in bronchial smooth muscle [35]. A decrease in miR‐133a expression is associated with the upregulation of the protein RhoA in bronchial tissue, which has been reported to induce smooth muscle contraction and contribute to small airway obstruction.

The prevalence of muscle loss in COPD ranges from 15% to 40% [S1, S2]. This decline in muscle mass has been extensively associated not only with the progression of COPD, marked by decline of lung function, but also with a cascade of adverse outcomes encompassing exercise intolerance, poor quality of life, and increased mortality [22, 23, 24, 25, 26]. Therefore, to minimize bias, we excluded participants with underlying chronic airway diseases from our analysis. Although the present study established a noteworthy association between decline in FEV_1_ and FVC concerning both MMI and rate of MMI decline in general population, considerable effect on AFO was not revealed. These findings might be attributed to unique characteristics of the study cohort. Unlike COPD cohorts, subjects enrolled in the KoGES cohort displayed distinctive traits. Subjects included in the KoGES cohort were younger than other COPD cohorts, with an average age of approximately 50 years. In addition, over 65% of the individuals in this cohort had never been smokers. These distinct features of the KoGES cohort underscore the potential influence of age and smoking history on observed relationships between muscle mass and AFO. In addition to aging, cigarette smoking contributes to a decrease in lung function with shared mechanisms such as oxidative stress, systemic inflammation, and induction of muscle loss and dysfunction [5, 26, 36]. Therefore, in a relatively young cohort population with a high proportion of never smokers, the risk of developing AFO might not have significantly increased during the 12‐year follow‐up period.

BMI calculated from weight and height is an easily measurable metabolic indicator. However, accuracy of BMI in reflecting true muscle mass is questionable, as it does not effectively distinguish between muscle and fat mass. Muscle loss does not always coincide with a reduction in adipose tissue [10, 14, ]. In addition, previous studies have shown a paradoxical relationship between overweight/obesity and COPD regarding not only mortality risk [S6−S8], but also lung function decline [S9, S10]. Therefore, there is a controversy over the suitability of BMI as an appropriate metabolic index in the context of COPD. BIA is another simple and easy tool that provides information on water distribution between intracellular and extracellular compartments and various body composition data, enabling the assessment of muscle mass, fat mass, and fat‐free mass [11]. The MMI is a simple index that can be derived from BIA measurement. In this study, while differences in fat mass were observed, fat free mass did not vary significantly across MMI tertiles. This finding suggests that MMI could be a valuable indicator of sarcopenia, as it may better reflect true muscle mass. Due to its convenience, BIA is widely used in the general population and chronic illness patients, and MMI has been reported to be associated with impaired functional capacity in COPD patients [S4‐S6, S10]. Additionally, muscle mass is not a static measure; it is a dynamic value that can change over time.

One of the main objectives of this study was to uncover the dynamic relationship between muscle mass and pulmonary function in the general population, recognizing the fluctuation in muscle mass with aging and the need to investigate longitudinal changes and their impact on lung function. Moreover, we not only evaluated FEV_1_ and FVC, but also assessed relationships between muscle mass and other lung function metrics including FEF25–75 that showed significant declines with implications for ongoing small airway obstruction. The KoGES database enabled us to analyse body composition and metabolic parameters across MMI tertiles uncovering notable associations with fat mass and metabolic syndrome. This supports the idea that muscle mass changes could influence metabolic health, which might be accompanied by lots of chronic respiratory illness.

The observed data showed that the decline rate of MMI was not directly associated with AFO. However, lung function metrics including FEV_1_, FVC, and FEF25–75 showed significant differences, decreasing at the fastest rate in the cT1 group, whereas the rate of decline was slower in cT2 and cT3 groups. Although we reported FEV_1_ declines of 42.9 mL/year, 38.1 mL/year, and 34.5 mL/year across the different MMI tertile groups, they were below the minimal clinically important difference (MCID) of 100 mL/year [37]. However, given that this study was conducted in a general population, even smaller differences could be clinically meaningful and have substantial impacts on health, considering lung function changes accumulate over several years to decades. Furthermore, there was a significant lower likelihood of exacerbation episodes in cT3 than in cT1. This raises important considerations regarding the meaning of variation of MMI over time in the context of lung function decline and respiratory symptom development. The differentiation in decline rate across these MMI underscores a potential progressive nature of lung function impairment, which may not necessarily be accompanied by overt AFO. Varying rates of decline in FEV_1_, FVC and FEF25–75 indicate that while airflow may not be obstructed per se, lung function is indeed compromised to varying degrees by MMI changes. Moreover, the risk of developing a wheezing event was significantly reduced in cT3 than in cT1. The presence of more respiratory symptoms, such as wheezing and dyspnoea, is associated with increased airflow obstruction, and wheezing has been identified as a potential predictor of small airway disease, with relative ratios of 2.9 (95% CI: 2.0, 4.3) in smokers and 3.3 (95% CI: 1.9, 5.8) in nonsmokers. [38]. This suggests a need for a personalized approach in monitoring and managing patients, taking into account exacerbation and lung function. In this context, the rate of MMI change could be a critical surrogate. Maintaining muscle mass might also be regarded as a tailored treatment and preventive strategy.

This study evaluated the effect of the rate of change in muscle mass on change in lung function through simple body composition measurement and follow‐up tests in a large general population cohort. Aging related muscle mass reduction might be a risk factor for the development of chronic respiratory disease. This alarms us to pay attention to patients' outcomes by using a simple method to assess sarcopenic status and possible intervention to improve muscle loss. However, this study has several limitations. Firstly, although the KoGES cohort is a general population cohort, it is confined to a specific geographical region. As regional specificity may raise concerns regarding diversity, there are potential challenges when attempting to extrapolate findings of this study to a wider population. Secondly, this study lacks information on distribution of muscle through imaging techniques such as computed tomography (CT). Absence of detailed muscle mass data limits a more comprehensive understanding of the relationship between muscle mass and lung function. Thus, a more in‐depth analysis of the relationship between distribution of muscle in the body and lung function was unavailable. Thirdly, we assessed pulmonary functions solely on pre‐BD values, which might not capture potential changes or variations in lung function after bronchodilation. Fourth, muscle strength was not included in the KoGES database. Although muscle loss is associated with decreased muscle strength and exercise tolerance [39, 40], a comprehensive approach to sarcopenia has not been obtained yet. Fifth, personal socio‐economic data were obtained by trained interviewers, but there is a potential for recall bias in the self‐reported data. Lastly, external validation was not possible due to the absence of other longitudinal cohorts with repeated measurements of lung function and body composition together. Additionally, this limitation restricts the generalizability of the study's findings.

## 5 Conclusions

This study highlights the association between the rate of MMI decline and lung function changes, as well as the risk of exacerbation of respiratory symptom. Out findings suggest that individuals with slower rates of MMI decline tend to experience a slower reduction in lung function and may have a lower risk of respiratory symptom exacerbation. This underscore the importance of monitoring MMI changes over time for respiratory health. However, further research is needed to better understand these relationships and to evaluate the potential long‐term benefits of interventions aimed at improving or maintaining muscle mass on respiratory health.

## Disclosure

None of the authors has any financial relationships with a commercial entity with an interest in the subject of this manuscript.

## Ethics Statement

Ethical approval was obtained from the Ethics Committee of Incheon St. Mary's Hospital, and the IRB number was OC23ZISI0033. The requirement for informed consent was waived by the Ethics Committee of Incheon St. Mary's Hospital.

## Conflicts of Interest

The authors declare no conflicts of interest.

## Supporting information


**Figure S1.** Flowchart of the study. COPD, chronic obstructive pulmonary disease; FEV_1_, forced expiratory volume in 1 s; FVC, forced vital capacity; PFT, pulmonary function test.

**Figure S2.** Scheme of MMI decline rate categories (cT1‐T3). The cut‐off values for the MMI rate of change corresponding to the tertiles are as follows: cT1: Below −2.102226, cT2: Between −2.102226 and 1.007333, and cT3: Above 1.007333. MMI, muscle mass index.

**Figure S3.** Longitudinal trend of lung function by MMI decline rate. (A) FEV_1_ (B) FVC (C) FEF25–75 (D) FEV_1_/FVC All changes in lung function were analysed with adjustments for age, sex, BMI, education and income level and smoking status. * vs. cT1 *p* < 0.05; ^#^ vs. cT3 p < 0.05 BMI, body mass index; FEF, forced expiratory flow; FEV_1_, forced expiratory volume in 1 s; FVC, forced vital capacity; MMI, muscle mass index.

**Figure S4.** Kaplan–Meier survival curves for the time to the first AFO, stratified by the rate of MMI decline. ^#^ *p* = 0.130; ***p = 0.384.

**Data S1.** Supporting Information.

**Table S1.** Biochemical and Body composition measurements by MMI categories.
**Table S2.** Time to AFO.
**Table S3.** Time to AFO (sensitivity analysis).
**Table S4.** Time to first exacerbation (sensitivity analysis).

**Data S2.** Supporting Information.

## Data Availability

Researchers interested in utilizing the KoGES epidemiological data can find access through online sharing platform (http://www.kdca.go.kr/research/KoGES/datasharing). Additionally, a guide for navigating the integrated dataset is provided within the online sharing system.
