# Supplementary figures and images for: Muscle Mass Index Decline as a Predictor of Lung Function Reduction in the General Population
Source: J Cachexia Sarcopenia Muscle. 2024 Dec 17;16(1):e13663. doi: 10.1002/jcsm.13663 (PMC11693984; doi:10.1002/jcsm.13663)

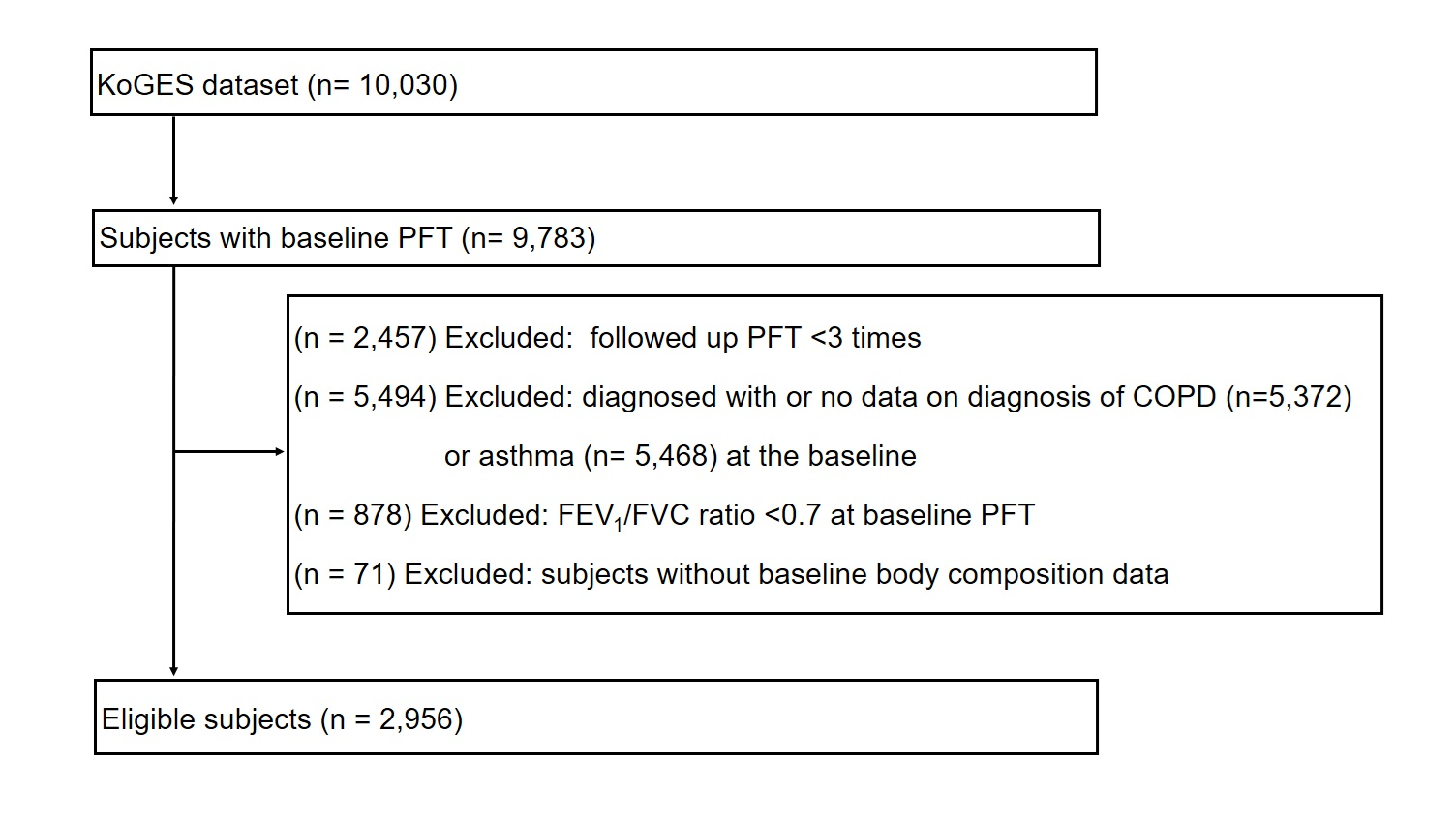

Supplement: Supplementary file 1 — Figure S1. Flowchart of the study. COPD, chronic obstructive pulmonary disease; FEV1, forced expiratory volume in 1 s; FVC, forced vital capacity; PFT, pulmonary function test. [file JCSM-16-e13663-s007.tiff]

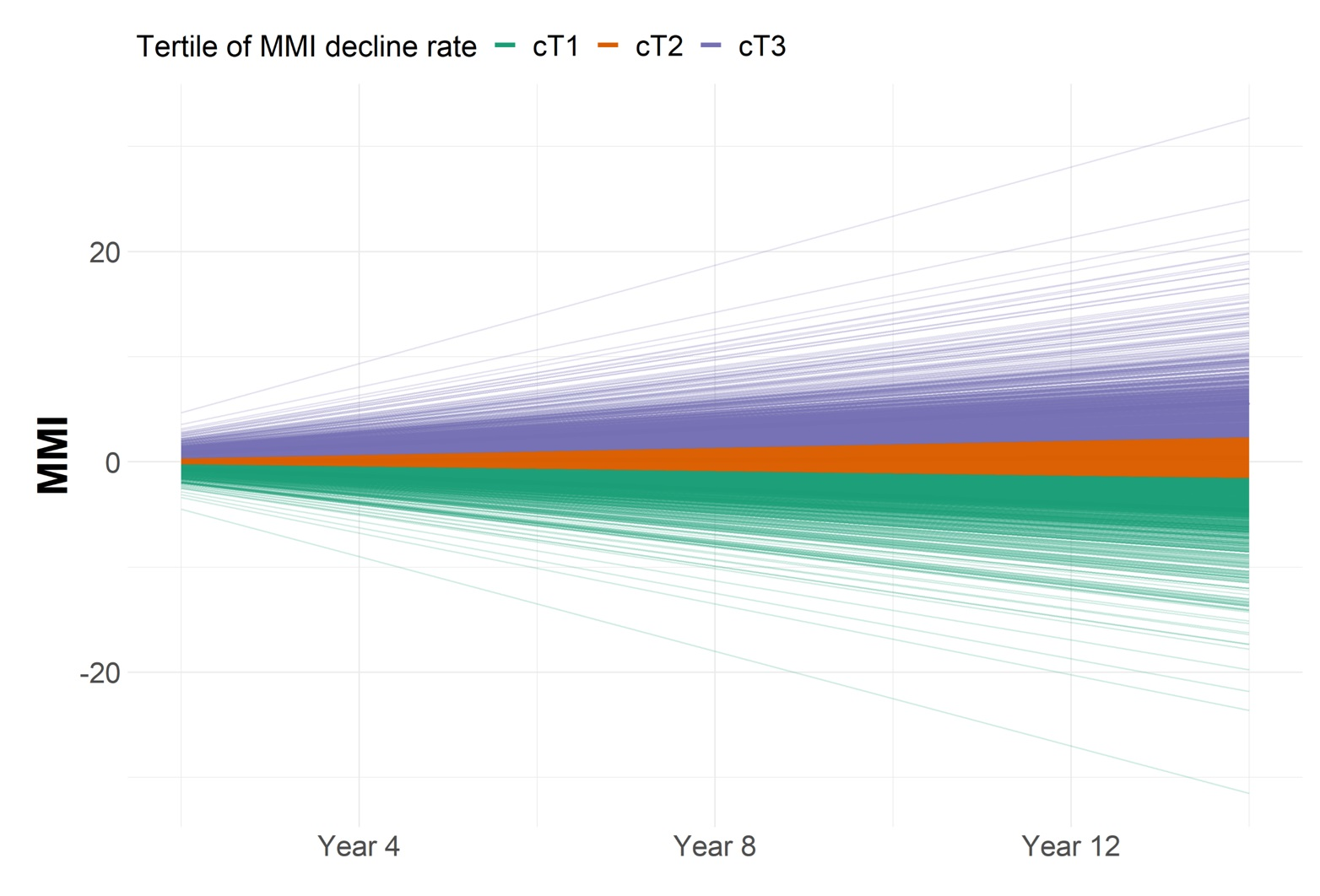

Supplement: Supplementary file 2 — Figure S2. Scheme of MMI decline rate categories (cT1‐T3). The cut‐off values for the MMI rate of change corresponding to the tertiles are as follows: cT1: Below −2.102226, cT2: Between −2.102226 and 1.007333, and cT3: Above 1.007333. MMI, muscle mass index. [file JCSM-16-e13663-s003.tiff]

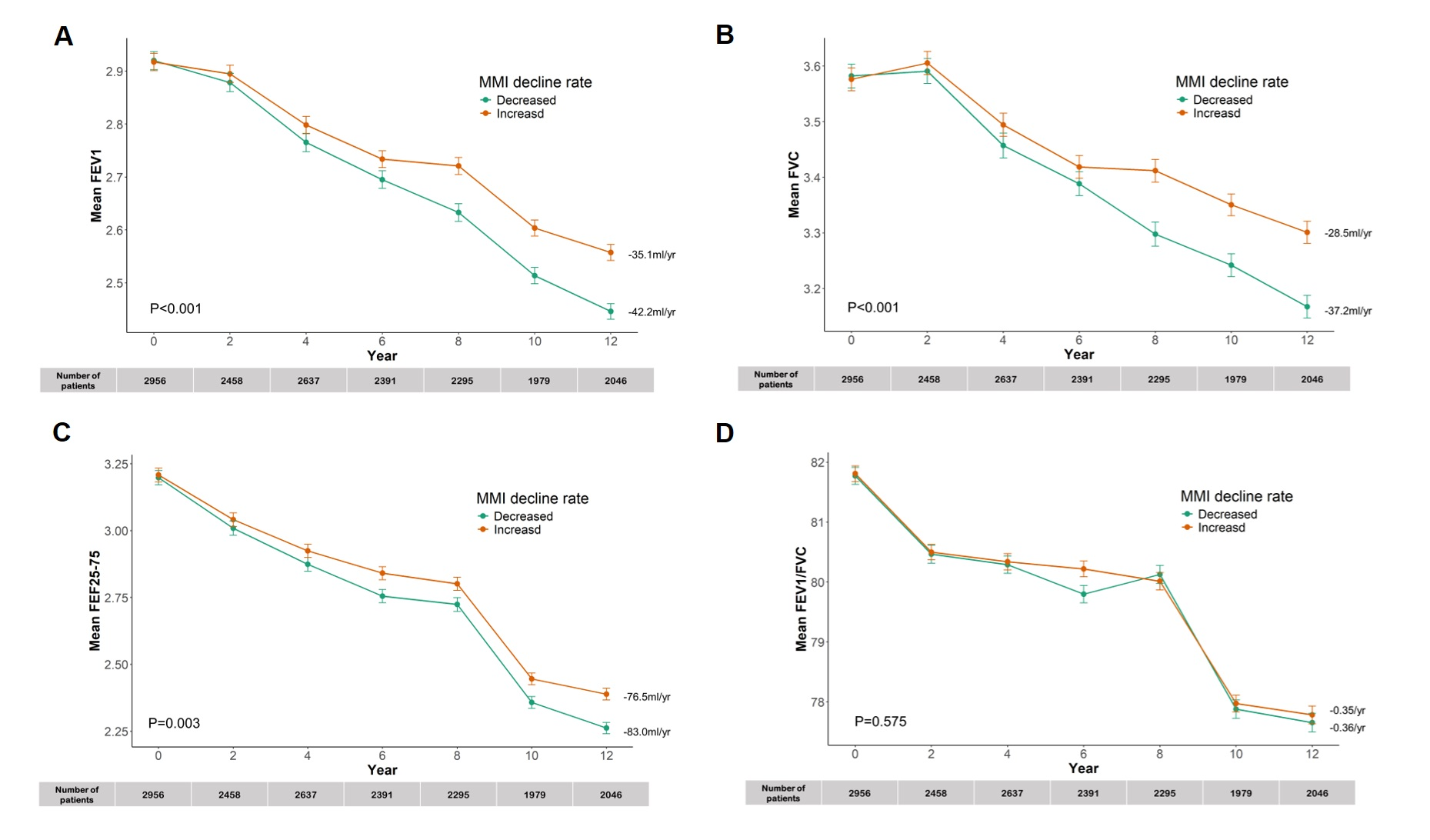

Supplement: Supplementary file 3 — Figure S3. Longitudinal trend of lung function by MMI decline rate. (A) FEV1 (B) FVC (C) FEF25–75 (D) FEV1/FVC All changes in lung function were analysed with adjustments for age, sex, BMI, education and income level and smoking status. * vs. cT1 p < 0.05; # vs. cT3 p < 0.05 BMI, body mass index; FEF, forced expiratory flow; FEV1, forced expiratory volume in 1 s; FVC, forced vital capacity; MMI, muscle mass index. [file JCSM-16-e13663-s002.tiff]

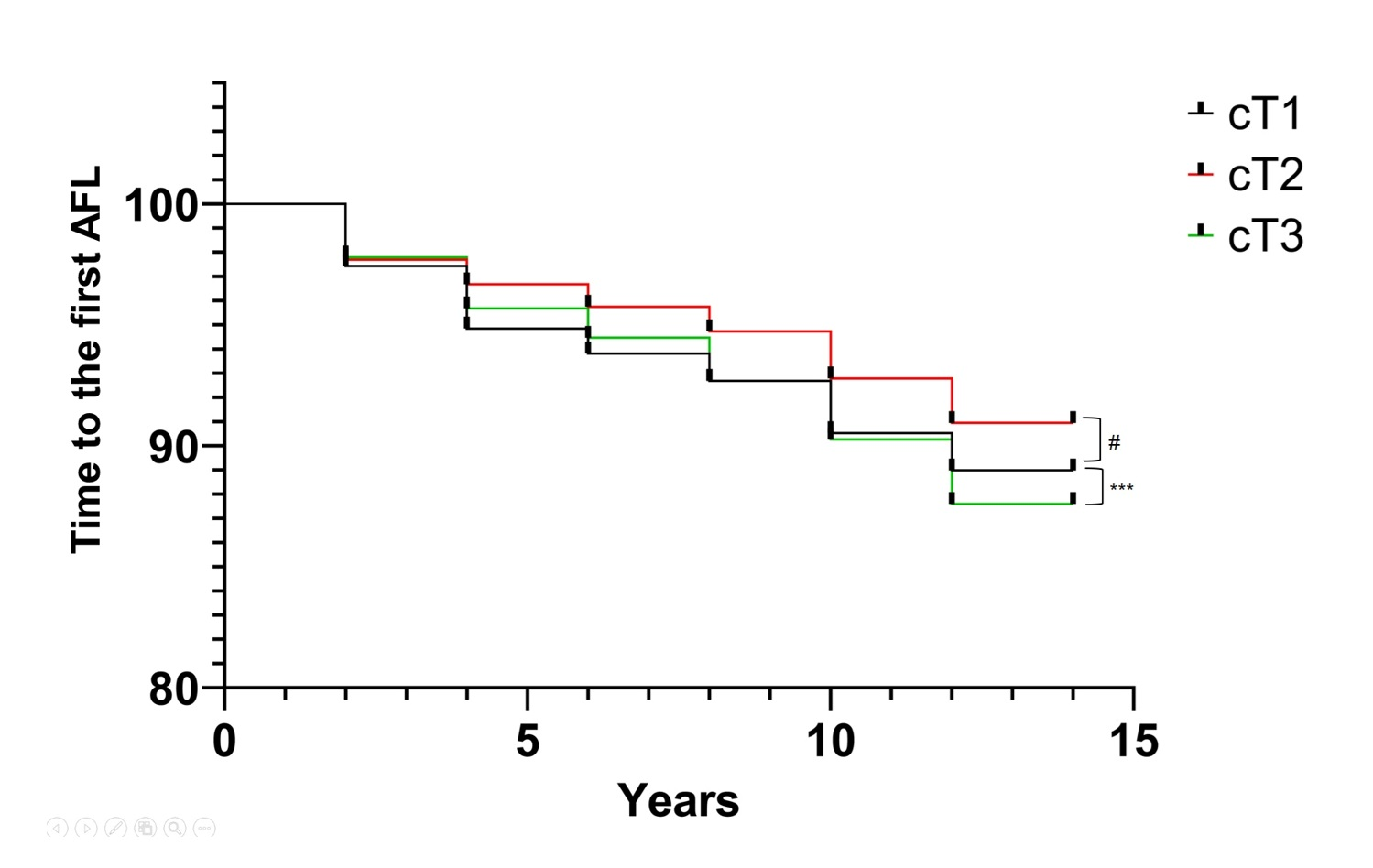

Supplement: Supplementary file 4 — Figure S4. Kaplan–Meier survival curves for the time to the first AFO, stratified by the rate of MMI decline. # p = 0.130; ***p = 0.384. [file JCSM-16-e13663-s006.tiff]
