# Supplementary material for: Muscle Mass Index Decline as a Predictor of Lung Function Reduction in the General Population
Source: J Cachexia Sarcopenia Muscle. 2024 Dec 17;16(1):e13663. doi: 10.1002/jcsm.13663 (PMC11693984; doi:10.1002/jcsm.13663)
Supplement: Supplementary file 6 — Table S1. Biochemical and Body composition measurements by MMI categories. Table S2. Time to AFO. Table S3. Time to AFO (sensitivity analysis). Table S4. Time to first exacerbation (sensitivity analysis). [file JCSM-16-e13663-s005.docx]

**"Online Supplementary data"**

**Table S1. Biochemical and Body composition measurements by MMI categories**

|  | **Overall (N = 2,956)** | | | |
| --- | --- | --- | --- | --- |
|  | T1  (N=941) | T2  (N=1,009) | T3  (N=1,006) | P for trend |
| Muscle mass (kg) | 43.6 ± 7.8 | 43.2 ± 8.1 | 42.8 ± 7.9 | 0.113 |
| Total Body water (L) | 31.9 ± 5.7 | 31.7 ± 6.0 | 31.4 ± 5.8 | 0.114 |
| Intracellular water (ICW, L) | 21.4 ± 3.9 | 21.2 ± 4.1 | 21.0 ± 4.0 | 0.205 |
| Extracellular water (ECW, L) | 10.6 ± 1.9 | 10.4 ± 1.9 | 10.3 ± 1.9 | 0.031 |
| Fat mass (kg) | 22.9 ± 4.3 | 17.8 ± 2.9 | 13.3 ± 3.4 | <0.001 |
| Fat free mass (kg) | 46.1 ± 8.2 | 45.8 ± 8.5 | 45.3 ± 8.3 | 0.114 |
| Percentage body fat (%) | 33.4 ± 5.5 | 28.4 ± 5.1 | 22.8 ± 5.7 | <0.001 |
| Body protein, kg | 11.6 ± 2.1 | 11.5 ± 2.2 | 11.4 ± 2.1 | 0.111 |
| BMI (kg/m2) | 27.6 ± 2.5 | 24.9 ± 2.0 | 22.6 ± 2.2 | <0.001 |
| Weight circumference ≥90 cm (male) or ≥85 cm (female) | 527 (56%) | 297 (29.4%) | 124 (12.4%) | <0.001 |
| Fasting glucose (mg/dL) | 89.5 ± 20.7 | 88.2 ± 23.4 | 84.3 ± 18.8 | <0.001 |
| Insulin or hypoglycemic agent use | 81 (8.6%) | 85 (8.4%) | 60 (6%) | 0.047 |
| Blood pressure |  |  |  |  |
| SBP ≥130 mmHg | 355 (37.7%) | 285 (28.3%) | 204 (20.3%) | <0.001 |
| DBP ≥85 mmHg | 396 (42.1%) | 313 (31.1%) | 240 (23.9%) | <0.001 |
| Antihypertensive medication | 279 (29.6%) | 177 (17.5%) | 121 (12%) | <0.001 |
| TG (mg/dL) | 189.0 ± 119.1 | 170.8 ± 110.9 | 136.8 ± 74.2 | <0.001 |
| Dyslipidemia medication | 16 (1.7%) | 7 (0.7%) | 3 (0.3%) | 0.003 |
| HDL cholesterol | 42.1 ± 8.9 | 42.7 ± 9.5 | 45.8 ± 10.0 | <0.001 |
| Metabolic syndrome* | 487 (51.9%) | 337 (33.4%) | 169 (16.8%) | <0.001 |

Data are presented as number (%) or mean ± SD

* Metabolic syndrome was defined as the presence of three or more of the followings ^1^: 1) waist circumference of ≥90 cm for males or ≥85 cm for females representing abdominal obesity defined by the Korean Society of Obesity ^2^; 2) hypertriglyceridemia, defined as a serum TG concentration of ≥ 150 mg/dL, or treatment for dyslipidemia; 3) low HDL, defined as a serum HDL cholesterol concentration <40mg/dL for males or <50 mg/dL for females; 4) high blood pressure, defined as a SBP ≥130 mmHg or a DBP of ≥85 mmHg, or treatment with antihypertensive agents; and 5) high fasting glucose level, defined as a fasting serum glucose level of ≥100 mg/dL or current use of antidiabetic medication.

BMI, body mass index; DBP, diastolic blood pressure; HDL, high-density lipoprotein; SBP, systolic blood pressure; TG, triglyceride

**Table S2. Time to AFO**

|  | **Hazard ratio (95% Confidence interval)** | | | | |
| --- | --- | --- | --- | --- | --- |
|  | **Crude HR** | **Model 1** | **Model 2** | **Model 3** | **Model 4** |
| **MMI decline rate** |  |  |  |  |  |
| cT1 | 1 (reference) | 1 (reference) | 1 (reference) | 1 (reference) | 1 (reference) |
| cT2 | 0.809  (0.615-1.064) | 0.830  (0.629-1.093) | 0.810  (0.613-1.070) | 0.820  (0.620-1.084) | 0.996  (0.653-1.518) |
| cT3 | 1.125  (0.863-1.466) | 1.106  (0.846-1.445) | 1.036  (0.789-1.359) | 1.052  (0.801-1.382) | 1.182  (0.764-1.830) |

Model 1: adjusted for age, sex, BMI, smoking status

Model 2: adjusted for age, sex, BMI, smoking status, physical activity, FEV_1_

Model 3: adjusted for age, sex, BMI, smoking status, physical activity, FEV_1_~~,~~ metabolic syndrome

Model 4: adjusted for age, sex, BMI, smoking status, physical activity, FEV_1_~~,~~ metabolic syndrome, respiratory symptoms (dyspnea, wheeze, cough, sputum), radiologic abnormalities (emphysema, interstitial lung abnormalities, bronchiectasis)

AFO, airflow obstruction; BMI, body mass index; FEV_1_, forced expiratory volume in 1s; MMI, muscle mass index

**Table S3.** **Time to AFO (sensitivity analysis)**

|  | **Hazard ratio (95% Confidence interval)** | | | | |
| --- | --- | --- | --- | --- | --- |
|  | **Crude HR** | **Model 1** | **Model 2** | **Model 3** | **Model 4** |
| **MMI decline rate** |  |  |  |  |  |
| Decreased | 1 (reference) | 1 (reference) | 1 (reference) | 1 (reference) | 1 (reference) |
| Increased | 1.002  (0.804-1.249) | 1.000  (0.800-1.250) | 0.965  (0.770-1.210) | 0.977  (0.779-1.225) | 0.864  (0.613-1.218) |

Model 1: adjusted for age, sex, BMI, smoking status

Model 2: adjusted for age, sex, BMI, smoking status, physical activity, FEV_1_

Model 3: adjusted for age, sex, BMI, smoking status, physical activity, FEV_1_~~,~~ metabolic syndrome

Model 4: adjusted for age, sex, BMI, smoking status, physical activity, FEV_1_~~,~~ metabolic syndrome, respiratory symptoms (dyspnea, wheeze, cough, sputum), radiologic abnormalities (emphysema, interstitial lung abnormalities, bronchiectasis)

BMI, body mass index; FEV_1_, forced expiratory volume in 1s; MMI, muscle mass index

**Table S4.** **Time to first exacerbation (sensitivity analysis)**

| **MMI decline rate** | **Hazard ratio (95% Confidence interval)** | | | |  |
| --- | --- | --- | --- | --- | --- |
|  | **Crude HR** | **Model 1** | **Model 2** | **Model 3** | **Model 4** |
| Wheezing | | | | |  |
| Decreased | 1 (reference) | 1 (reference) | 1 (reference) | 1 (reference) | 1 (reference) |
| Increased | 0.752  (0.627-0.902) | 0.757  (0.630-0.909) | 0.748  (0.622-0.900) | 0.752  (0.624-0.905) | 0.676  (0.511-0.892) |
| Wheezing or dyspnea | | | | |  |
| Decreased | 1 (reference) | 1 (reference) | 1 (reference) | 1 (reference) | 1 (reference) |
| Increased | 0.752  (0.627-0.902) | 0.757  (0.630-0.909) | 0.748  (0.622-0.900) | 0.752  (0.624-0.905) | 0.676  (0.511-0.892) |

Model 1: adjusted for age, sex, BMI, smoking status

Model 2: adjusted for age, sex, BMI, smoking status, physical activity, FEV_1_

Model 3: adjusted for age, sex, BMI, smoking status, physical activity, FEV_1_^,^ metabolic syndrome

Model 4: adjusted for age, sex, BMI, smoking status, physical activity, FEV_1_~~,~~ metabolic syndrome, radiologic abnormalities (emphysema, interstitial lung abnormalities, bronchiectasis)

BMI, body mass index; FEV_1_, forced expiratory volume in 1s; MMI, muscle mass index

**Figure legends**

**Figure S1. Flowchart of the study**

COPD, chronic obstructive pulmonary disease; FEV_1_, forced expiratory volume in 1s; FVC, forced vital capacity; PFT, pulmonary function test

**Figure S2. Scheme of MMI decline rate categories (cT1-T3).** The cutoff values for the MMI rate of change corresponding to the tertiles are as follows: cT1: Below -2.102226, cT2: Between -2.102226 and 1.007333, and cT3: Above 1.007333.

MMI, muscle mass index

**Figure S3.** **Longitudinal trend of lung function by MMI decline rate****.** (A) FEV_1_ (B) FVC (C) FEF25-75 (D) FEV_1_/FVC

All changes in lung function were analyzed with adjustments for age, sex, BMI, education and income level and smoking status.

^*^ vs cT1 p<0.05; ^#^ vs cT3 p<0.05

BMI, body mass index; FEF, forced expiratory flow; FEV_1_, forced expiratory volume in 1s; FVC, forced vital capacity; MMI, muscle mass index

**Figure S4. Kaplan-Meier survival curves for the time to the first AFO, stratified by the rate of MMI decline.**

^#^: p=0.130; ^***^: p=0.384
