# Supplementary material for: Muscle Mass Index Decline as a Predictor of Lung Function Reduction in the General Population
Source: J Cachexia Sarcopenia Muscle. 2024 Dec 17;16(1):e13663. doi: 10.1002/jcsm.13663 (PMC11693984; doi:10.1002/jcsm.13663)
Supplement: Supplementary file 7 — Data S2. Supporting Information. [file JCSM-16-e13663-s001.docx]

**SUPPLEMENTARY REFERENCES**

[S1.](https://www.ncbi.nlm.nih.gov/pmc/articles/PMC11003524/)  Schols AM, Broekhuizen R, Weling-Scheepers CA, Wouters EF. Body composition and mortality in chronic obstructive pulmonary disease. Am J Clin Nutr. 2005;82(1):53-9.

[S2.](https://www.ncbi.nlm.nih.gov/pmc/articles/PMC11154800/)  Vestbo J, Prescott E, Almdal T, Dahl M, Nordestgaard BG, Andersen T, et al. Body mass, fat-free body mass, and prognosis in patients with chronic obstructive pulmonary disease from a random population sample: findings from the Copenhagen City Heart Study. Am J Respir Crit Care Med. 2006;173(1):79-83.

[S3.](https://pubmed.ncbi.nlm.nih.gov/35982215/) Machado FVC, Spruit MA, Groenen MTJ, Houben-Wilke S, van Melick PP, Hernandes NA, et al. Frequency and functional translation of low muscle mass in overweight and obese patients with COPD. Respir Res. 2021;22(1):93.

S4. van de Bool C, Gosker HR, van den Borst B, Op den Kamp CM, Slot IG, Schols AM. Muscle Quality is More Impaired in Sarcopenic Patients With Chronic Obstructive Pulmonary Disease. J Am Med Dir Assoc. 2016;17(5):415-20.

S5. Martínez-Luna N, Orea-Tejeda A, González-Islas D, Flores-Cisneros L, Keirns-Davis C, Sánchez-Santillán R, et al. Association between body composition, sarcopenia and pulmonary function in chronic obstructive pulmonary disease. BMC Pulm Med. 2022;22(1):106.

S6. de Blasio F, Scalfi L, Di Gregorio A, Alicante P, Bianco A, Tantucci C, et al. Raw Bioelectrical Impedance Analysis Variables Are Independent Predictors of Early All-Cause Mortality in Patients With COPD. Chest. 2019;155(6):1148-57.

S7. Landbo C, Prescott E, Lange P, Vestbo J, Almdal TP. Prognostic value of nutritional status in chronic obstructive pulmonary disease. Am J Respir Crit Care Med. 1999;160(6):1856-61.

S8. Yamauchi Y, Hasegawa W, Yasunaga H, Sunohara M, Jo T, Takami K, et al. Paradoxical association between body mass index and in-hospital mortality in elderly patients with chronic obstructive pulmonary disease in Japan. Int J Chron Obstruct Pulmon Dis. 2014;9:1337-46.

S9. Wu Z, Yang D, Ge Z, Yan M, Wu N, Liu Y. Body mass index of patients with chronic obstructive pulmonary disease is associated with pulmonary function and exacerbations: a retrospective real world research. J Thorac Dis. 2018;10(8):5086-99.

S10. Sun Y, Milne S, Jaw JE, Yang CX, Xu F, Li X, et al. BMI is associated with FEV(1) decline in chronic obstructive pulmonary disease: a meta-analysis of clinical trials. Respir Res. 2019;20(1):236.
